# Long-term efficacy of percutaneous tibial nerve stimulation for faecal incontinence and a new approach for partial responders

**DOI:** 10.1007/s10151-022-02711-z

**Published:** 2022-10-12

**Authors:** M. Bosch-Ramírez, L. Sánchez-Guillén, M. J. Alcaide-Quirós, M. M. Aguilar-Martínez, M. Bellón-López, A. López Delgado, F. López-Rodríguez-Arias, A. Muñoz-Duyos, X. Barber-Valles, A. Arroyo

**Affiliations:** 1 Department of General Surgery, Colorectal Unit, Elche University Hospital, University Miguel Hernández of Elche, Camino de la Almazara 11, 03203 Elche, Alicante Spain; 2 Department of General Surgery, Colorectal Unit, Mútua Terrassa University Hospital, University of Barcelona, Terrassa, Barcelona Spain; 3 Center for Operations Research, University Miguel Hernández of Elche, Elche, Alicante Spain; 4 Joint Research Unit on Statistical Methods in Health Sciences UMH-FISABIO, University Miguel Hernández of Elche, Elche, Spain

**Keywords:** Percutaneous tibial nerve stimulation, Faecal incontinence, Neuromodulation, Partial response, Long term efficacy

## Abstract

**Background:**

The aim of the present study was to evaluate the long-term efficacy of percutaneous tibial nerve stimulation (PTNS) for patients with faecal incontinence (FI) refractory to conservative treatment. Secondary aims were to identify predictors of response and validate new treatment pathways for partial responders.

**Methods:**

A prospective, interventional study was carried out in a specialist defecatory disorder unit from a university hospital between January 2010 and June 2017 on patients > 18 years old with FI refractory to conservative treatment. Thirty-minute PTNS sessions were performed in three phases (weekly, biweekly and monthly) up to a year, with clinical reassessment at 3, 6, 12 and 36 months.

Patients were classified as optimal responders when their pretreatment Wexner score decreased > 50%; partial responders when it decreased 25–50%; and insufficient responders if it decreased < 25%. Only optimal and partial responders progressed into successive phases.

**Results:**

Between 2010 and 2017, 139 patients (110 women, median age 63 years [range 22–82 years]) were recruited. After the first phase, 4 patients were optimal responders, 93 were partial responders and 36 were insufficient responders. At 6 and 12 months, 66 and 89 patients respectively were optimal responders, with an optimal response rate of 64% at the end of treatment. A total of 93.3% patients with a partial response initially finally became optimal responders. Furthermore, at 36 months, 71.9% of patients were still optimal responders without supplementary treatment, although their quality of life did not improve significantly. Baseline Wexner scores ≤ 10 and symptom duration < 1 year were identified as predictive factors for positive responses to PTNS.

**Conclusions:**

Patients undergoing PTNS for 1 year following this protocol had optimal long-term responses. PTNS sessions for up to 1 year in patients who were partial responders prevents a high percentage of them from needing more invasive treatments, and maintains long-term continence in patients who were optimal responders.

**Supplementary Information:**

The online version contains supplementary material available at 10.1007/s10151-022-02711-z.

## Introduction

The prevalence of faecal incontinence (FI) is 11–15% [1, 2] and increases progressively with age [3]. Even though FI can decrease patients’ quality of life (QoL) due to social stigma and embarrassment, only 10–30% of sufferers seek medical advice [4].

The initial method of managing FI is conservative and based on dietary changes, pelvic floor exercises and biofeedback, but more invasive treatments are often necessary [5]. Sacral nerve stimulation (SNS) has been shown to be an effective alternative for treating FI, providing direct, chronic low-voltage stimulation of the sacral root, with good long-term results [6]. However, it requires surgical insertion and has high associated costs [7, 8]. Tibial nerve stimulation, applied with transcutaneous or percutaneous (PTNS) electrodes over the posterior tibial nerve (L5 to S3 nerve roots), provides peripheral stimuli to treat both urinary and faecal incontinence [9]. It is a safer, less invasive and cheaper alternative to SNS for FI.

PTNS has been shown to be effective in some patients, improving FI symptoms and QoL in the short term. The CONFIDeNT trial [10], which compared PTNS to sham stimulation found that PTNS did not confer significant clinical benefit after 3 months of treatment. Few randomized trials have attempted to compare SNS and PTNS, concluding that in the short term (6 months) both SNS and PTNS confer some clinical benefit for patients with FI [11]. Therefore, short- and mid-term outcomes (24 months) have been reported [10, 12–14] but there is a need to assess long-term efficacy.

Moreover, several PTNS regimens have been reported, ranging from daily to monthly sessions, performed transcutaneously or percutaneously at outpatient clinics or home-based. All of these factors complicate a comparison between regimens and results.

The primary aim of this study was to evaluate the long-term efficacy of PTNS for patients diagnosed with FI refractory to conservative treatment. Secondary aims included identifying predictors of responses and a new approach for partial responders.

## Materials and methods

A prospective, interventional study was carried out in a specialist defecatory disorder unit at Elche university hospital between January 2010 and June 2017. Long-term was defined as a 36-month period of time, as previously described by Thinn [8] and considered for long-term evaluation for SNS [6].

Patients ≥ 18 years old were eligible for inclusion if they had a diagnosis of FI (one or more FI episodes per week for more than 6 months) refractory to conservative treatment. The exclusion criteria were anatomical abnormalities that required surgery (congenital sphincter defects, ano-vaginal cloacae and complex perianal fistulas according to ASCRS classification [15]), injuries affecting ≥ 180° of the external anal sphincter, previous treatment with PTNS, unavailability to attend regularly outpatient clinic and major psychological or psychiatric disorders.

Data were recorded prospectively and evaluated by two members of the aforementioned unit (MJAQ, ALD) including patients’ medical history, symptom duration, bowel habit (21-day diary) [16], faecal urgency and severity of FI, as estimated by the Wexner score and by FI episodes per week: mild (1–6 episodes per week), moderate (7–11 per week), and severe (≥ 12 per week). Functional tests (endoanal ultrasound and anorectal manometry), were also performed. QoL was assessed with the Faecal Incontinence Quality of Life scale (FIQLs) [17] and the Rapid Assessment Faecal Incontinence Score (RAFIS) [18].

**Fig. 1:**
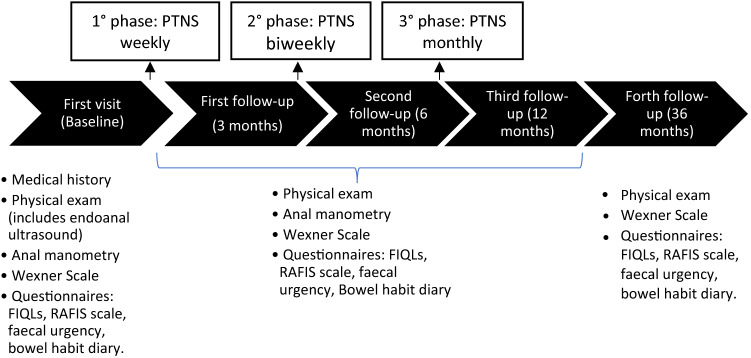
PTNS process. *PTNS* percutaneous tibial nerve stimulation, *FIQLs* faecal incontinence quality of life scale, *RAFIS* rapid assessment faecal incontinence score

Evaluations were performed at baseline, 3, 6, 12 and 36 months as shown in Fig. 1. The Wexner score, FIQLs and faecal urgency were evaluated at each follow-up visit. The RAFIS score and the bowel habit diary were also recorded at 12 and 36 months, and manometric measurements were repeated at 12 months.

Patients lost to follow-up were considered in intention-to-treat analysis, and all complications related to the procedure were recorded during the study. There were no substantial modifications to schedule nor evaluation of the patients during the 7.5 years of this study.

### PTNS protocol

PTNS was administered by 30-min sessions performed in three phases. Sessions were performed weekly for 3 months in the first phase. During second phase sessions were performed biweekly, for 3 months. Lastly, sessions were performed monthly in the third phase, which lasted 6 more months. Therefore, treatment was administered up to a year.

Stimulation was performed by members of the unit (MBR, MMA, MBL) with the technique described by Hotouras using an “Urgent® PC stimulator” (Uroplasty, Minnetonka, MN, USA) [19].

At the end of each phase patients were classified as follows: optimal responders, defined as patients with a reduction > 50% in the initial Wexner score; partial responders, defined as a reduction between 25 and 50%; or insufficient responders, defined as a reduction < 25%.

Patients categorized as optimal or partial responders were considered potential long-term beneficiaries of PTNS and progressed to the successive treatment phases. In the next phases all these patients, received the same treatment, with the goal of maintaining the clinical and QoL benefits in the optimal responder and to achieve optimal response in patients with previous partial response. Insufficient responders (< 25%) were offered other therapeutic options such as SNS.

### Statistical analysis

The data were analysed using SPSS software, version 24 (IBM-SPSS; Chicago, IL, USA), and R software (2020) with the ISLR package to obtain the classification and regression trees (CARTs).

The quantitative results are expressed as the mean (median for the non-normally distributed data) and standard deviation. The categorical variables are expressed as the number of cases and percentages.

The sample size was calculated using an 80% for the power and 5% for significance for the expected incidence of the disease. After the selection process and the loss of patients during the follow-up the post-hoc power was 71%.

Associations between the quantitative and normally distributed variables were analysed using Student’s *t-*test, while the categorical variables were analysed using the *χ*^2^ test. *P* < 0.05 was considered to be statistically significant.

Classification and regression tree (CART) analysis involves a nonparametric decision tree and can efficiently segment populations into meaningful subgroups. This type of analysis was used to identify potential predictors of success among variables including the Wexner score, proctologic and obstetric parameters and symptom duration. To avoid misclassification, the pruning strategy was conducted [20].

## Results

A total of 139 patients (110 women, median age 63 years [range 22–82 years]) diagnosed with FI refractory to medical treatment were enrolled in the study. The demographic characteristics and comorbidities of the study population are described in Table 1. Fifty-nine patients (42.5%) were diagnosed with FI between 1 and 5 years after symptom onset, the majority with 2 to 3 normal bowel movements per day (22.1%). Some associated symptoms included urinary incontinence (29.8%), sexual dysfunction (6.4%) and pelvic pain (3.5%).

**Table 1:** Demographic and examination data

Historic morbidity	*N* (%)
Age, years*	63(22–82)
Sex (women/men)	110 (79.1%)/29 (20.9%)
Heart disease risk factors	
HTN	46 (33.8%)
DM	25 (18.4%)
Radicular disease	16 (11.5%)
Gastrointestinal disorders	
IBS	3 (2.2%)
Crohn’s disease	4 (2.9%)
Ulcerative colitis	2 (1.5%)
Cancer	
Colorectal	6 (4.4%)
Prostatic	2 (1.5%)
Previous surgery	
Pelvic surgery	33 (24.3%)
Sphincteroplasty	4 (2.9%)
Hysterectomy	25 (18.4%)
FI-postsurgical	49 (35.0%)
Anal pathology	
Rectocele	3 (2.2%)
Haemorrhoids	17 (12.5%)
Fistula	14 (10.8%)
Fissure	13 (9.6%)
Rectal prolapse	3 (2.2%)
Obstetric disorders	
Vaginal tear	38 (27.9%)
Dystocic birth	14 (10.3%)
Episiotomy	63 (46.3%)
Postpartum incontinence	4 (2.9%)
≥ 3 childbirth	22 (16.2%)
Vaginal rectocele	3 (2.3%)
Vaginal prolapse	7 (5.1%)
IF—post-partum relation	9 (6.4%)
Rectal examination	
Vaginal rectocele	3 (2.2%)
Rectocele	2 (1.5%)
Cystocele	5 (3.6%)
Scars	21 (15.3%)
Haemorrhoids	3 (2.2%)
Sphincter hypotonia	7 (5.1%)
Sphincter normal	29 (21.2%)

*HTA* hypertension, *DM* diabetes mellitus, *IBS* irritable bowel syndrome, *FI* faecal incontinence

*Median (range)

The rectal examination findings were normal in 28 (21.2%) patients, and 7 patients (5.1%) had hypotonic sphincters. Perineal scars were present in 21 patients (15.3%), and 10 (7.3%) patients had celes that were not considered appropriate for surgery. Endoanal ultrasound at baseline showed that 49 patients (35.2%) had no external anal sphincter defect, while 48 patients (34,6%) had a defect of < 90°, and 42 (30.2%) had defects of 90–180°.

### Clinical outcomes (Fig. 2)

**Fig. 2:**
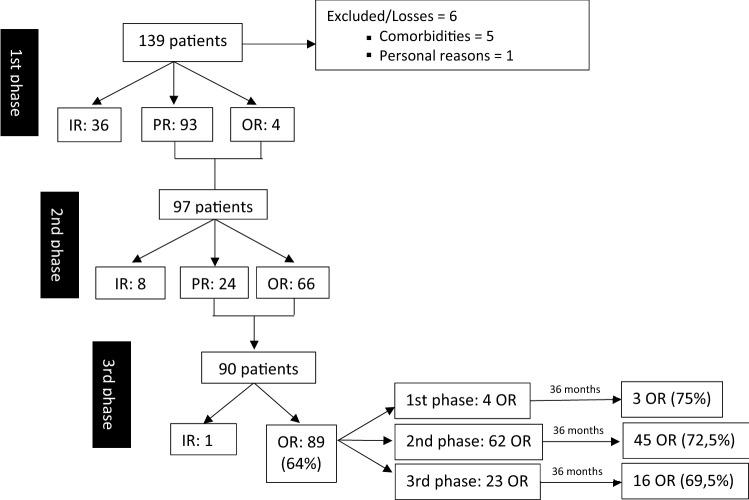
Clinical outcomes. *OR* optimal responders, *PR* partial responders, *IR* insufficient responders

At the 3-month follow-up, 4 (2.9%) patients were optimal responders, 93 patients (69%) were partial responders, and 36 patients did not improve after treatment (insufficient response)). Therefore, 97 patients were allowed to progress to the second phase of treatment.

At 6 months, the four patients who had been optimal responders continued to respond positively. Of the initial 93 patients with partial response, most became optimal responders (*n* = 62), and 24 remained partial responders.

The third and final phase was conducted for all optimal responders (4 from the first phase and 62 from the second phase) and partial responders (*n* = 24). The 66 optimal responders maintained an optimal response and 23 partial responders were classified as optimal responders at the 12-month follow-up. Only 1 patient was classified as an insufficient responder in that phase.

Hence, at the end of the treatment, optimal response was observed in a total of 89/139 (64.0%) patients, and (93.3%) of the patients initially classified as partial responders succeeded to achieve optimal response.

Patients’ progression is described in Fig. 2. At the 36-month follow-up, all optimal responders (*n* = 89) were revaluated, and 64 (71.91%) patients still had an optimal response without supplementary treatment. Specifically, 3 of the 4 patients who with an optimal response after the first phase of treatment continued to show benefit at the 36-month follow-up. Of the 62 patients with optimal response after the second phase, 45 patients maintained continence and 16 patients of the 23 with an optimal response after the third phase continued to show benefits at the final evaluation. The differences between groups were not statistically significant.

No major complications or side effects were observed. Six patients were lost to follow-up due to pulmonary and oncological comorbidities and personal reasons; these patients were considered patients with an insufficient response.

### Wexner score (Table 2)

**Table 2:** Wexner score and bowel habit diary results

Wexner (median) 25–75			Bowel habit diary		
		*p*	Mild (%)	Moderate (%)	Severe (%)
Baseline	11 (7–14)	*p* < 0.001	57.7	28.9	12.4
3 months	11 (5.5–15)		68	28	4
6 months	6 (2–9)		78.7	17	2.1
12 months	6 (3.5–10)		79.3	20	0.7
36 months	4 (1–8)		79.5	20	0.5

Severity groups according to the bowel habit diary: mild: 1–6; moderate: 7–11; severe: > 12

The median Wexner score decreased significantly from 11 (range 7–14) at baseline to 6 (range 2–9) at the 6-month follow-up (*p* < 0.001), and this magnitude of decrease was the largest observed. The scores continued to decrease to the end of the treatment, with a score of 6 (range 3.5–10) at 12 months. At the 36-month follow-up, after 2 years without additional treatment, the median Wexner score with previous optimal response was still 4 (range 1–8).

### Severity of incontinence (bowel habit diary) (Table 2)

Most patients (57.7%) had mild incontinence at baseline. While 17 patients (12.4%) showed severe incontinence at baseline, this rate decreased to four (0.5%) at the end of the study. Patients gradually turned from moderate and severe incontinence to mild incontinence (79.3%) at the end of treatment, and kept improving at 36 months reassessment (79.5%).

### RAFIS score (Table 3)

**Table 3:** Rapid Assessment for Faecal Incontinence Sore (RAFIS), and faecal urgency results

	RAFIS	Faecal urgency				
		< 1 min	1–5 min	5–10 min	> 10 min	*p*
Baseline	4.46 (± 2.67)	94 (67.7%)	29 (21.3%)	8 (5.5%)	8 (5.5%)	*p* < 0.001
3 months	3.82 (± 2.40)	61 (44%)	50 (36%)	17 (12%)	11 (8%)	
6 months	6.78 (± 2.13)	37 (37.7%)	27 (28.4%)	9 (8.8%)	24 (25.1%)	
12 months	6.07 (± 3.57)	16 (18.2%)	17 (18.4%)	6 (6.3%)	51 (56.3%)	
36 months	5.91 (± 1.10)	4 (4.35%)	4 (4.37%)	1 (1.48%)	80 (89.8%)	

*RAFIS* Rapid Assessment Faecal Incontinence Score

Prior to treatment, the mean RAFIS score was 4.5 (± 2.7). The score gradually increased to 6.1 (± 3.6) at the end of treatment (*p* = 0.16). However, the RAFIS score decreased to 5.9 (± 1.1) at the latest evaluation.

### Faecal urgency (Table 3)

A total of 94 (67.7%) patients could not delay defecation for more than 1 min at diagnosis, and 29 (21.3%) could delay defecation for 1–5 min. Eighth patients (5.5%) could delay defecation for 5–10 min, and 8 (5.5%) could delay defecation for more than 10 min.

The percentage of patients with severe faecal urgency (< 1 min) rapidly decreased from 94 patients (67.7%) to 61 (44%) at the first follow-up and even improved during the following phases of treatment. At the 6-month follow-up, twenty-four patients (25.1%) could delay defecation for more than 10 min, which was even higher at the 12 and 36-month follow-up (56.3% and 89.8%, respectively). These improvements were statistically significant in all phases (*p* < 0.001).

### Anal manometry

At diagnosis, the mean maximum resting pressure (MRP) was 31.8 mmHg (SD ± 18.4), and the maximum squeezed pressure (MSP) was 55 mmHg (SD ± 26.9). At the 6-month follow-up, the MSP remained stable at 59.1 mmHg (SD ± 27.8; *p* > 0.05), and the MRP increased to 35.2 mmHg (SD ± 15.9; *p* = 0.04).

### Quality of life (FIQLs) (Table 4)

**Table 4:** Faecal Incontinence Quality of Life scale (FIQLs)

FIQLs				
	Lifestyle	Behaviour	Depression	Embarrassment
Baseline	27 (± 9.8)	18 (± 8.5)	18 (± 7.5)	5 (± 9.8)
3 months	25 (± 8.9)	22 (± 9)	20 (± 6.9)	7 (± 4.6)
6 months	36 (± 7.3)	23 (± 6.8)	25 (± 4.5)	8.5 (± 3)
12 months	33 (± 7.8)	24 (± 8)	24 (± 4.9)	10 (± 2.6)
36 months	34.5 (± 7)	24 (± 7.5)	25 (± 5)	10.5 (± 2.7)
*p*	0.724	0.781	0.296	0.028

The data obtained from the QoL questionnaires showed that “embarrassment” was the domain that was rated worst by patients at the moment of diagnosis. At the 3-month follow-up, improvements were observed in behaviour and depression domains. At the 6-month follow-up, the lifestyle domain was particularly relevant, with a mean score of 36 (previously 27 and 25). At the 12-month follow-up, “embarrassment” was the domain with the largest improvement (5 points vs. 10 points), and the change was statistically significant (*p* = 0.03).

### CART (Fig. 3)

**Fig. 3:**
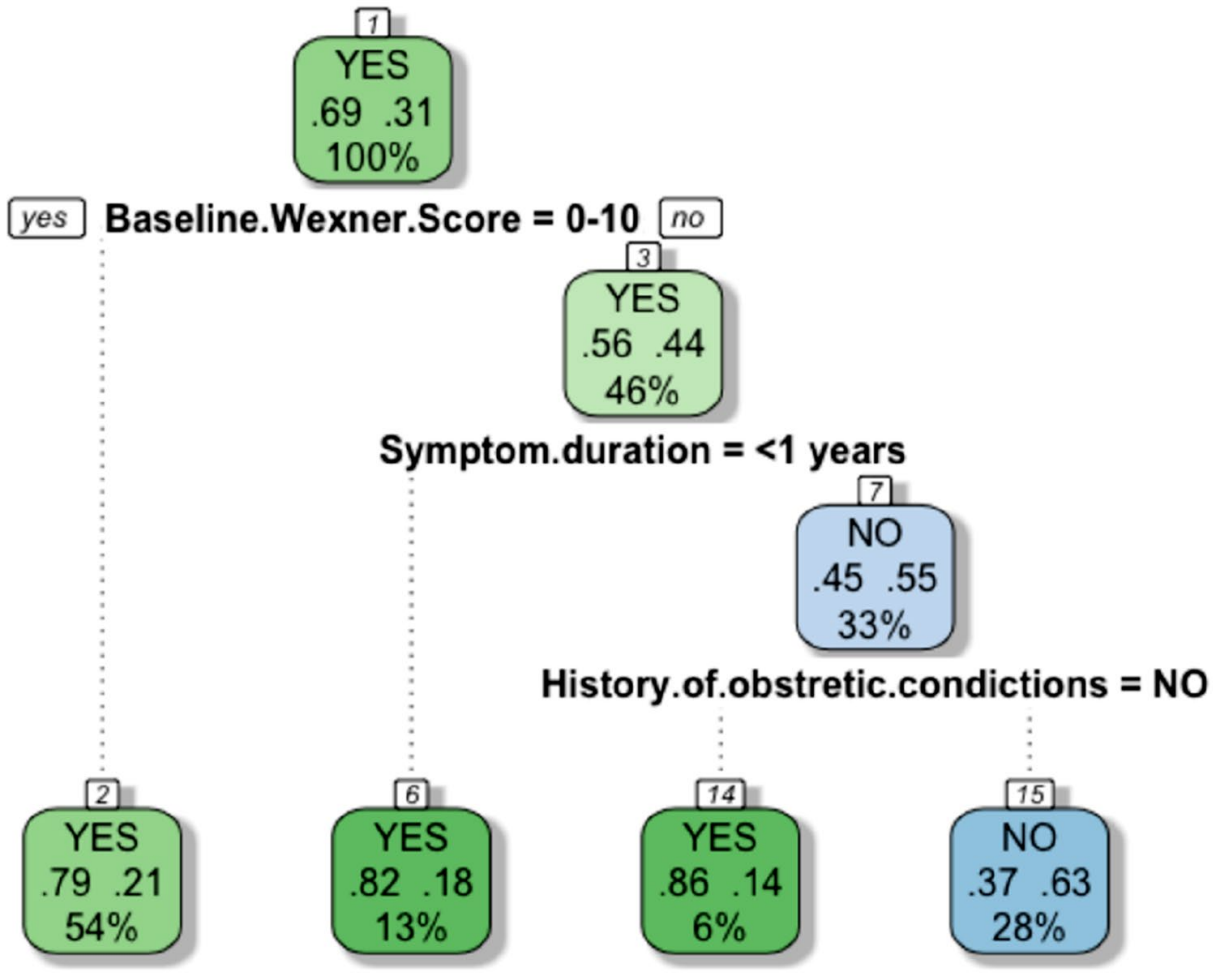
CART diagram. *CART* classification and regression tree

The CART procedure allowed the investigators to generate a tree containing the baseline Wexner score, symptom duration and obstetric history as predictive factors for PTNS success.

The results showed that a baseline Wexner score ≤ 10 suggests a high probability (79%) of obtaining a long-term optimal response. For a baseline Wexner score > 10, if symptom duration was ≤ 1 year, it is highly likely (82%) that the patient will exhibit optimal response in the long term. In contrast, if the baseline Wexner score is > 10, associated with symptom duration > 1 year and a history of obstetric conditions, the chance of the patient not exhibiting an optimal response is > 60%.

## Discussion

Since the first report on FI successfully being treated by using a needle electrode was published in 2003 [21], successful treatment of FI using PTNS has been observed in 60–70% of patients [9]. Queralto [22] reported an even higher success rate of 80% in the short term. Our results of 64% efficacy using the Wexner score were more modest, although still statistically and clinically significant. No previous study has considered partial response to be predictive of optimal response in the long term. We have demonstrated that over 90% of patients with a partial response will eventually have an optimal response if treatment is continued.

Our study is unique in testing potential predictors of success with a CART diagram. Baseline Wexner scores ≤ 10 and symptom durations < 1 year were revealed to be strong independent predictors for a good response, whereas Wexner scores > 10, symptom duration > 1 year and obstetric history were predictors of non-response. This finding might be useful for targeting PTNS and offering alternative treatment options (such as SNS) to patients without these predictors.

In 2010, Govaert [23] suggested that PTNS maintenance sessions are needed for long-term continence. Soon after, Hotouras [14] demonstrated that additional ‘top up’ therapy at 6-month intervals improved efficacy and maintained the benefits of PTNS over time. However, after a 2 year follow up, De La Portilla [13] found retreatment unnecessary, describing some improvement in FI severity beyond treatment phase, despite no ‘top up’ therapy. In our study, once an optimal response was observed, additional maintenance sessions were performed for up to 1 year. The time of an optimal response had no statistically significant impact on long-term efficacy. In line with the De La Portilla study we observed that the long term Wexner score fell despite no further treatment. This suggests that ‘top up’ therapy does more than simply maintain continence, and actually enhances efficacy. Further randomised assessment is needed.

The frequency of PTNS regimens is controversial. Thomas [24] compared daily vs*.* twice weekly stimulation. Subsequently, several regimes have been proposed [25]. Peña [26] reported significant benefits for weekly sessions performed for 3 months and biweekly sessions performed for 3 months when the patients responded optimally, as in our study. However, Hotouras [14, 19] and Govaert [23] administered additional sessions at 2 weeks, 4 weeks and 8 weeks for those responding to the initial 12 weekly sessions, with a maximum follow-up period of 26 months [14]*.* In our previously described schema, we mixed most relevant regimens, and all partial and optimal responders were treated equally, including weekly sessions for 3 months, biweekly sessions for 3 months, and monthly sessions performed for 6 more months. Therefore, the earlier the patient reached the optimal response, the more “top-up” sessions were administered. Subsequently, a new standardized follow-up at 36 months was introduced and was essential for determining long-term effectiveness.

Our regime of PTNS resulted in the severity of incontinence decreasing significantly, defined as mild by 80% of patients using the 3-week bowel habit diary. This level of incontinence did not deteriorate through the rest of the study period. Regarding faecal urgency, 67.1% of patients were initially unable to delay defecation for more than 1 min; by the end of the study, 56.2% of the patients were capable of delaying defecation for more than 10 min (*p* = 0.001). Similar findings were reported by Peña [26], with a significant variation in the ability to delay defecation from 2 to 11 min after treatment. Anal manometry showed statistically significant results regarding improvements in resting pressure with PTNS. These findings were consistent with those of a previous study in our unit [27] but inconsistent with other randomized trials [28], where only a significant increase in the maximum squeeze pressure at 3 months was found. However, it is important to remark that, although this change was statistically significant, the clinical relevance is questionable.

Undoubtedly FI has a profound effect on QoL but the effect is difficult to quantify. With the FIQLs, different domains are assessed allowing a global evaluation of FI [17]. We observed a mild improvement in QoL after PTNS, but embarrassment was the only domain with statistically significant improvement in the long term. Why an improvement in the Wexner score did not translate into improvement in all QoL domains remains unclear. Consistent with other studies [29], we found embarrassment the worst rated QoL domain. Any significant change was therefore more likely to be observed in this domain. Significant change may have been observed in other domains if there were a bigger sample size. Similarly, the RAFIS score improved after PTNS but the improvement was not significant again possibly due to sample size.

Our PTNS treatment regime appears effective, is easily scheduled and cheap. It can be performed as an outpatient procedure, allowing patients to continue their day-life activities and work and, as a result, improving compliance. Moreover, it does not alter pelvic anatomy, and therefore does not exclude subsequent invasive techniques for those that fail to respond.

Our study has several limitations. The main limitation is the absence of a control group for comparison. Additional randomized controlled trials including other techniques, larger sample sizes and longer follow-up periods are clearly required. Another limitation is the heterogeneity of the participant group, which complicates comparisons. Additionally, patients gaining OR only after final phase of treatment (3^rd^ phase) were not administered “top-up” sessions, which might lead to worsening in continence results in future reassessment. Defining a cut off sphincter injury in ≥ 180º for excluding PTNS as treatment may be controversial, lower cut off could be considered in the future according to daily practice. QoL data collection in a hospital environment with a practitioner at hand rather than at home might have reduced patient misinterpretation. A larger sample size and more frequent follow-up appointments should also be considered in future studies.

## Conclusions

Our results suggest that continued PTNS sessions in patients who show a partial response to an initial course of treatment may improve efficacy and avoid the need for alternative invasive therapy. CART diagram results highlighted the importance of raising awareness of, detect and treat FI within 1 year of symptom onset, as early treatment has been proven to be useful for observing long-term optimal response.

## Supplementary Information

Below is the link to the electronic supplementary material.

Supplementary file1 (PDF 142 KB)
